# Exploring Iraqi people’s perception about early marriage: a qualitative study

**DOI:** 10.1186/s12905-022-01980-y

**Published:** 2022-09-29

**Authors:** Abubakir M. Saleh, Samir M. Othman, Kameran H. Ismail, Nazar P. Shabila

**Affiliations:** 1 Department of Community Medicine, College of Medicine, Hawler Medical University, Erbil, Kurdistan Region Iraq; 2 Department of Nursing, Faculty of Nursing, Tishk International University, Erbil, Iraq; 3 Department of Medical Laboratory Sciences, Catholic University in Erbil, Erbil, Kurdistan Region Iraq

**Keywords:** Marriage, Erbil, Perception, Attitude, Qualitative research, Qualitative approach

## Abstract

**Background and objective:**

Early marriage is prevalent in Iraq, but the factors related to this behavior, especially people's attitudes about it, have not been well studied. It has been proven that early marriage seriously threatens young girls' lives and health. This study aimed to explore the perception of people about early marriage in the Iraqi Kurdistan region.

**Methods:**

A qualitative method was employed to carry out this study in 2020. Data were collected through 16 focus group discussions in different health centers in Erbil, Iraq. Each focus group included 8–12 participants who had the experience of early marriage. The transcripts of the focus groups were analyzed through the six methodological activities of Van Manen. The trustworthiness of the data was confirmed by using qualitative data evaluation criteria.

**Results:**

Different themes and subthemes regarding the ideal age of marriage, attitudes toward marriage, reasons for early marriage, advantages and disadvantages of early marriage, and practice of early marriage in the community emerged from focus group discussions. Most participants identified early marriage as inappropriate social behavior that should not be practiced. Reasons for early marriage included poor economic status, protection of girls and boys, low educational level, and respect for old traditions of the community. Divorce and violence against women were the main disadvantages of early marriage.

**Conclusion:**

There is a generally negative attitude towards early marriage, and the practice's disadvantages and health and psychological consequences are well recognized in Iraqi Kurdistan Region. However, different social, traditional, economic, educational, and religious factors encourage early marriage in the region. Further research is recommended in other governorates in the area to have a more precise and representative idea about the topic.

## Introduction

Early marriage, or child marriage, is prevalent and can affect children as young as nine. Many of these young girls might reach puberty at their husbands’ houses. Sometimes these very young girls get married to husbands who are as old as their grandfathers [1]. According to UNICEF, child marriages are referred to as marriage under 18 [2]. Early marriage harms girls as it steals their childhood innocence and turns them prematurely into adults. At this sensitive age of marriage, they are susceptible to diseases and are vulnerable to domestic violence. Early marriage often robs a girl or child of educational opportunities [1].

Early marriages occur all over the world. About 12 million girls marry under the age of 18 annually [3]. 95% of these girls are from middle- and low-income countries [4], where one out of every nine girls marries before age 15, and one out of every three marry before age 18 [5]. Worldwide, 1 in 7 marriages occurs before 15 years of age [6]. Accordingly, around 48% or approximately 10 million girls in Southern Asia, 42% of girls in Africa, and29% of girls in Latin America and the Caribbean are married by 18 [7].

Child marriage takes place for a variety of reasons like dropping out of school girls, poor socioeconomic status of the girl's family, religion, protection of the family honor, as well as some specific marriage customs such as cradle betrothal, cousin marriage, and berdel (exchange of brides between two families) lead to the early marriage of girls in these areas [2].

The early marriage rate has unprecedentedly increased in Iraq. Baghdad, the capital of this country, has the highest number of occurrences [8]. In fact, because of its traditional background and religious beliefs, Iraq is predisposed to the phenomenon of high rates of early marriage. Thus, Iraqi girls are increasingly obliged to get married at an early age. It is reported that before the 1990s, the father gave girls to a stranger or a relative, who regarded his daughter as a gift to offer to the suitor.

According to the Iraq family socioeconomic survey in 2007, around 21% of girls married before 19 years old, which was much higher than the 15% rate in 1997 and 2004. Another study in 2011 showed that around 5% of girlsbelow15 years and 22% of those below18 years were married in Iraq.

The ministry of women’s affairs in Iraq issued a warning in 2010 stating that girls aged 15–18 years were two times more likely to die from pregnancy and childbirth compared with women aged 20–24 years [9]. However, the causes and determinants of early marriage and people's attitudes about this important behavior affecting women's health are not well understood [10]. Attention to this warning from the Ministry of Health and other international organizations is one of the essential responsibilities of researchers, planners, and health policymakers in Iraq. In this regard, this study conducted to explore the perception of people about early marriage in the Iraqi Kurdistan region.

## Method

### Study design

This study used a qualitative approach and a conventional content analysis method. Qualitative content analysis is an appropriate and coherent method used to analyze text data for a better experience and knowledge of the phenomenon. One of the essential features of qualitative research is that it allows for close attention to be paid to the participant's perspective and understanding of the world through their eyes.

### Study setting

The study setting included different health centers in Erbil governorate, includingKasnazan, Binaslawa, Daratoo, and Briaty.

#### Time of the study

April to December 2020.

#### Study population

The study population consisted of people who lived in the Erbil governorate, Kurdistan region, Iraq. The study participants were selected through purposive sampling because they have experience with this topic and could contribute more to the study. The participants accessed the authorities of the different health centers in the Erbil governorate.

The inclusion criteria were people having a history of early marriage in the past year, living in the Kurdistan region, and willing to participate in the study.

### Data collection

Required data were collected through 16 focus group discussions that were carried out in different health centers in Erbil province (Brayaty, Kasnazan, Benaslawa, Daratoo). The focus group discussion method provided real-life data through discussions with the men and women. This method offers room for more in-depth exploration during data collection. Each focus group included 8–12 participants during the morning time. Each session lasted around 45 min—topic guide questions were used in the focus group discussion. Semi-structured discussions were carried out using an interview guide (Table 1). Participants were asked to talk freely on issues raised and questions asked during the focus groups on early marriage. The focus groups were tape-recorded and transcribed verbatim. Then, the transcripts were analyzed into different themes. The researchers continued the focus group until the conceptual saturation was reached, which resulted in no further new categories and themes from focus group no. 14. Still, the researchers continued the focus for up to 16 to get more assured [11, 12].

**Table 1:** Focus group questions

1. What is the ideal age for marriage?
2. How do you define early marriage?
3. What is your opinion about early marriage?
4. What are the reasons for early marriage?
5. What are the advantages of early marriage?
6. What are the disadvantages of early marriage?

### Data analysis

The transcripts of the focus groups were analyzed through the six methodological activities of Van Manen [13], as shown in Table 2.

**Table 2:** Methodological activities of Van Manen

#	Van Manen’s methodical activities	The researchers’ activities
1	Turning to the nature of lived experience	People's (Men and women) lived experiences of early marriage can be vital for planning and policymaking
2	Investigating experience as we live it	An adult person aged more than 18 years with a history of early marriage
3	Reflecting on the essential themes which characterize the phenomenon	Thematic analysis was employed
4	Describing the phenomenon through the art of writing and rewriting	A phenomenological text was created through writing and rewriting techniques
5	Maintaining a solid and oriented relation to the phenomenon	The themes were discussed concerning the phenomena
6	Balancing the research context by considering parts and whole	The transcripts and the extracted themes were compared and studied closely

The initial coding's accuracy and precision were verified using a set procedure and competent subject-matter experts. The present study's strength was evaluated using the Lincoln and Guba criteria. Participants assisted in the continuing verification and coding of data to ensure its acceptability for accuracy and authenticity. Two research team members independently decoded the data to assess dependability and reached a strong consensus. Additionally, significant consideration and enough effort were put into the data collection, implementation, and encoding processes. Three outside researchers with expertise in sociology, qualitative research, and early marriage obtained data in the area of transferability [14].

### Trustworthiness

Trustworthiness is the level of adequacy or soundness in qualitative studies [15]. Ensuring a qualitative study's trustworthiness involves essential steps such as describing data analysis and justifying the reliability of the gathered data [16]. In this study, providing the trustworthiness involved considering the field experts' comments, creating a good relationship and obtaining the women's trust, using suitable time and place for the interviews, and reading the transcripts several times. Moreover, the researchers' long-term field activity helped ensure their reliability.

### Ethical consideration

Informed consent was obtained from all the participants after explaining all the study details. Confidentiality of the participants was kept by keeping the recording of the focus group discussion only with the research team with no access to other people.

## Results

One hundred forty-two people participated in this study. Eighty-six of them (60.6%) were female, forty-three (30.3) had secondary school qualifications, and fifty-seven (40.1%) were in the age group (30–39) years (Table 3).

**Table 3:** Sociodemographic characteristics of the participants

Age group	20–29	24	16.9%
	30–39	57	40.1%
	40–49	45	31.7%
	50–59	16	11.3%
Gender	Male	56	39.4%
	Female	86	60.6%
Educational level	Illiterate	32	22.5%
	Primary school	39	27.5%
	Secondary school	43	30.3%
	College and higher education	28	19.7%
Residence	Urban	76	53.5%
	Sub-urban	52	36.6%
	Rural	14	9.8%
Total		142	100%

The sixteen focus groups provided a broad representation of views and sufficient saturation. Different themes and subthemes regarding the ideal age of marriage, attitude toward marriage, reasons for early marriage, advantages and disadvantages of early marriage, and practice of early marriage in the community emerged from focus group discussions (Table 4).

**Table 4:** Theme and subtheme of focus group discussion

1. Ideal age of marriage
1.1 Difference between the age of men and women
2. Definition of early marriage
3. Attitude toward early marriage
4. Reasons for early marriage
4.1 Poor economic status
4.2 Girls’ and boys' protection
4.3 Dropping out of school by girls
4.4 The tradition of the community
4.5 New technology, internet, mobile phone, and Facebook
4.6 Imitation of other
4.7 Parent mentality and thinking
4.8 Conflict in the family
4.9 Religion
4.10 Being an immigrant
5. Advantages of early marriage
5.1 Protection of boys and girls
5.2 Having kids at a younger age
5.3 Healthier kids
5.4 Younger society
6. Disadvantages of early marriage
6.1 Divorce
6.2 Adverse effects on body and mind
6.3 Financial problems
6.4 Violence against women and suicide
6.5 Cheating
6.6 Failure to continue education
6.7 Feeling bored after a short period

**1. Ideal age of marriage**

On the ideal age of marriage, most of the participants stated that 25 years old is the perfect age for marriage for both men and women. Some of the participants gave responses like this:

The ideal age for marriage for males and females is 25 years because this age is mature. They are more mature; Twenty-five years is the ideal age for marriage because they are mature at that time and they know the meaning of life. M, 35 Y

Few participants stated that the age should be even more than twenty-five years old: *The age of marriage should be above twenty-five years because they know the responsibilities of life better. *F, 36 Y

Only one participant mentioned that the marriage should be as early as possible: *Boys and girls should marry as early as possible because of religious reasons.* M, 46Y

**1.1 Difference between the age of men and women**

Some participants mentioned that at the time of marriage, the girls should be younger than boys: “The *ideal age is twenty-two years for females and twenty-five years for males.”;* “*The ideal age is twenty for females and twenty-five for males; females should be younger than males."* F, 35 Y

**2. Definition of early marriage**

The majority of the participants defined marriage as marriage before eighteen years. A few described it before fifteen: “*Early marriage is the marriage before eighteen years.";“Marriage before 15 years."* M, 28 Y.

**3. Attitude toward early marriage**

The majority of the participants said that it is a bad thing and should not be done. Joint statements were: *“It is not good because the female or male knows nothing about marriage life, they are immature, and have no life experiences”;* "*Not good because they are immature both physically and mentally*." F, 42 Y.

Few participants mentioned that early marriage depends on the situation and circumstances of the marriage. Sometimes it is good and successful. Examples:“*Depend on the situation and circumstances of the marriage. We have a very successful early marriage.”* F, 37 Y.

**4. Reasons for early marriage**

Different reasons for early marriage were mentioned by various participants in the focus group discussions.

**4.1 Poor economic status**

Most participants mentioned that poor economic status is the main reason for early marriage, especially if the family has many girls and wants to marry them early to get rid of them. Common statements were:

Sometimes families are poor and have many girls, so they arrange an early marriage to get rid of them. M, 38 Y

Poor economic status to get rid of girls, especially when a family has many girls. F, 42 Y

**4.2 Protection of girls and boys**

Many participants mentioned that the main reason for early marriage in our community is to protect boys and girls from doing unacceptable behaviors by our culture and to make them stable in their families. Examples of comments made:

To protect boys and girls from doing bad things and to make them stable. F, 37 Y

Current circumstances are unsuitable, so parents want to marry their kids earlier to avoid mistakes. M, 39 Y

**4.3 Dropping out of school by girls**

Some participants mentioned that when girls leave their education, their parents marry them earlier. Example of comments made:

When a girl leaves the school, the family will prepare marriage for her. F, 28 Y

**4.4 The tradition of the community**

Some participants mentioned that our community's tradition is one reason for early marriage. Example of comments made:

Old traditional mentality of the community which encourages early marriage is one of the reasons. M, 30 Y

**4.5 New technology, internet, mobile phone, and Facebook**

Another reason for early marriage mentioned by some participants was the latest technology, mobile phone, and Facebook because this will make communication much more accessible than before and people can find each other easily. Examples of comments made:

New technology, mobile phone and internet make relationship easier between boy and girls which may result in early marriage. M, 29 Y

**4.6 Imitation of others**

Imitation is another reason for early marriage mentioned by some participants. Example of comments made:

Sometimes a girl imitates her friends when many of her friends marry at an early age, so she would like to marry early. F, 33 Y

**4.7 Parent mentality and thinking**

Few participants mentioned that parents' thinking and mentality are another cause of early marriage in our community. Example:

Old and traditional thinking of the parents. They think that boys and girls should marry early. M, 28 Y

**4.8 Conflict in the family**

A few participants also mentioned that conflict in the family orbad relationships among different family members are other reasons for early marriage. Examples of comments made:

Sometimes early marriage results from tribe conflict in which they give their small daughter to another family to solve the problem. F, 52 Y

Sometimes girls want to marry as soon as possible because of the problems in the family. M, 37 Y

**4.9 Religion**

Two of the participants mentioned that religion is the reason for early marriage. Example

Our religious advice is to prepare marriage for our children as soon as possible. M, 42 Y

**4.10 Being an immigrant**

Only one participant mentioned that the increasing number of immigrants in our region nowadays is another reason for early marriage in our society. Example:

Increasing immigrants in our region, especially from other parts of Iraq and Syria, make the immigrant families marry their daughter early to get rid of them because of financial problems. M, 35 Y

**5. Advantages of early marriage**

The majority of participants mentioned that there are no advantages to early marriage, but a few participants mentioned some benefits like:

**5.1 Protection of boys and girls**

The boy and girl will be far from bad things and become stable, and they will go to their home. F, 39 Y

**5.2 Having kids at a younger age**

They will have kids younger, and the kids grow with them to help them. M, 37 Y

**5.3 Healthier kids**

Their kids will be healthier because parents are young and healthy. F, 32 Y

**5.4 Young society**

The society will be young and active, unlike western countries where they suffer from a lack of young and active people. M, 32 Y

**6. Disadvantages of early marriage**

**6.1 Divorce**

Most participants mentioned that this type of marriage is immature and faces many problems which end in divorce, and the kids will be the primary victims. Examples of joint statements made:

The early marriage might be ended with the divorce. This is because of immature mind of the early marriage couples. M, 38 Y

Many problems including divorce and sometimes they have kids. So, the kids will be the victim of this marriage. F, 43 Y

Husband and wife have no experience in life so that any simple problem may lead to divorce. Sometimes even hearing a few words from a friend may lead to a divorce. This is same for boy and girl. M, 31 Y

**6.2 Negative effects on the body and mind**

Some participants mentioned that this type of marriage is unhealthy and affects the girl physically and psychologically. Examples:

Early marriage is not healthy, might affect the females' reproductive organs when pregnant. F, 29 Y

The early marriage has a psychological effect on couples, especially females. F, 33 Y

**6.3 Financial problems**

Few participants said that because couples are usually unemployed and have no income, they often face many financial problems and will be dependent on their parents. Examples of comments:

Because most of them are unemployed and do not have their income, they live with their parents, which creates many problems in the family. M, 41 Y

They could not manage themselves economically and might depend on parents. F, 45 Y

**6.4 Violence against women and suicide**

Some participants mentioned that early marriage ended with violence against the wife and even sometimes suicide of the wife. Examples of comments:

Continuous conflict between them and sometimes the husband uses violence against the wife. F, 33 Y

Sometimes this type of marriage ends with wife suicide. F, 41 Y

**6.5 Cheating**

Two participants mentioned that the couple cheats on each other in early marriage, i.e., doing a relationship with another person rather than their husband or wife. Example:

Cheating on each other is common in this type of marriage. M, 48 Y

**6.6 Failure to continue their education**

One participant mentioned that if the couples are students, they will not be able to continue their education:

If the couple is students, they cannot continue their education because of different problems they face during the marriage. M, 52 Y

**6.7 Feeling bored after a short period**

Another participant mentioned that after a few years, the couples feel bored with each other:

After a few years of marriage. They may feel bored and dislike each other, especially if there is a difference in the level of education between them. M, 38 Y

## Discussion

This study explored people’s perception about early marriage in the Iraqi Kurdistan region. Most study participants stated that 25 years old is the ideal age for marriage for both men and women. Studies from other settings have shown different results. A study from Indonesia revealed that 74% of the student participants considered 19–24 years of age the ideal age range for marriage for women, and 68% considered 25–30 years the perfect age range for men to marry [17]. In a study among Syrian refugees, half of the respondents believed that the average ideal age of marriage for girls is less than 18 [18]. Another study in India revealed a significant difference between the ideal age of marriage in rural and urban areas. The perfect age in urban areas was 21 years, while it was 18–20 years in rural areas [19].

In the current study, the participants mentioned different reasons for early marriage, such as poor economic status of the girl's family, protection of girl and boys, low educational level, the tradition of the community, imitation, parent mentality and thinking, conflict in the family, religion and increasing number of immigrants in the community.Various and complex reasons lead to early marriage, but most are related to motivation among the marriage partners or their parents. For instance, among the Masaai in Kenya, some parents believe that marrying off their daughters at an early age will secure their futures, as opportunities for women to gain a livelihood outside marriage are scarce [20]. In most cases, early marriage is associated with poverty. In some developing countries, such as Bangladesh, female children are seen as a burden on the economic resources of family units; in such situations, parents marry off their daughters to have one less mouth to feed [21]. Also, family honor plays a significant role in parents and children's decision to marry early, especially for young girls. Studies from South Asia and Sub-Saharan Africa suggest that many families are concerned about their daughter's innocence and worry about premarital pregnancy, believing it will affect their honor and bring shame to the family. Hence, early marriage seems like a suitable solution to these problems in the context of their cultural constructs [22].

Social and family trend is another main reason for early marriage in some settings, such as Africa. A survey conducted in Ethiopia showed that many rural people think that a late adolescence non-married girl represents a failure and disgrace to the family. They think this will particularly compromise the status of the girl's father, as he will be regarded as a failure when his daughter is not married by late teens [23]. In the remote Baitadi district in western Nepal, the astrological birth chart of a girl holds the keys to her future [24]. In another study done in Aljazeera state in Sudan, 86.9% mentioned culture as a reason for early marriage [25]. Those citing culture might relate this to the belief that women's opportunities for marriage decrease with age due to a preference among men for younger wives or poverty-related issues causing people to want to have more children. In some cases, this is related to girls being pushed to marry early to preserve family honor.

In the current study, a low educational level was recognized as an important reason for early marriage. Another study from Iraq showed that education is significantly associated with child marriage. It showed that 26.5% of women aged 20–24 with no education and 19.6% with primary education were married or in the union at age 18, compared to only 10.3% of women with secondary education or higher [4]. In a UNICEF study in 42 countries, women aged 20–24 who had attended primary school were less likely to marry by age 18 than those who had not. Therefore, education may be one way to prevent child marriage [26]. Also, in Nepal, women face gender discrimination in access to education. They get married because they do not attend school. For instance, the overall literacy rate of Dalits is 34.8 for females. Only 31.6% of girls are enrolled in school [27]. Also, In Iraq in 2006, 19.1% of women were married between 20 and 24 years in rural areas compared to 15.9% of women in urban areas [4]. Another study from rural Egypt revealed that once girls begin to menstruate should avoid talking to boys and change their daily activities too [28].

Protecting the girl was another important reason for early marriage in our study. Also, in another study, families in Burundi refugee camps marry their daughters off as early as possible to protect their honor. Other pressures might encourage early marriage in societies under stress. Early marriage might be prevalent in places with a high incidence of domestic violence, trafficking, rape, sexual slavery, and abduction of children [3]. A study from India revealed that half of the girls were withdrawn from school at the time of first menses, mainly to get married as soon as possible. This is because menarche is thought to be a sign of getting married or due to the disgrace and danger of having a pubescent girl in public while unmarried [28]. A study from Somalia showed that early marriage of girls was aimed at preventing premarital sex [28]. In some African countries, men seek to marry a young virgin for fear of HIV infection [3]. Sometimes, parents think that the early marriage of their daughters protects them from HIV/AIDS [28].

Poor economic status was another reason for early marriage, as identified by our study participants. Studies from Iraq revealed that early marriage is increasing in response to poverty resulting from the post-Gulf War sanctions [3]. Previous studies in Kurdistan [29] and Iran [30] have identified poverty as the main reason for getting married early. Still, only 16.40% of our respondents cited this. Evidence from another study done in Kurdistan [31] in 2018 showed that childhood marriage increased during financial hardships in Iraq, and in 1997 a total of 15%of marriages were to females aged below 18 years. In Iraq, girls were pushed to marry men from other tribes as blood compensation, “Diyyah” or “blood money". This form of marriage compensates for a crime committed by the bride's brother or family/tribe member against another family or tribe [28].

Poor economic status is also a recognized reason for early marriage in other settings. For example, marriage in Ethiopia is intended to build coalitions or agreements between families and contributes to the parents’ status. When a girl's family is poor, marriage alleviates the financial cost of raising the girl and offers some financial reward from the bride price [28]. In Sub-Saharan Africa, the bride’s family in some traditional societies might get cattle from the groom, or his family, as the bride price for the girl. A study from Bangladesh showed that poor parents are convinced to part with daughters through marriage promises. A UNICEF study in West Africa showed that economic hardship is increasing early marriage, even among the people who do not practice it usually. The war and militarization in Afghanistan have resulted in a rise in forced marriages of young girls [3].

Religion is another reason for early marriage identified by the current study's participants. A study in Iran reported that some adolescents marry to meet their sexual needs [32]. Religious and cultural beliefs may be background factors for such rationales for early marriage, but religion was the last thing that came to our participants' minds. Conversely, another study conducted in Iran [33] showed that religion is one of the most common causes. Over a fifth (22.6%) of our respondents believed that marriage would complete half of the religion, reflecting a hadith of the Prophet Mohammed (peace and blessings upon him), who said, "Whoever marries has achieved one half of one's religion." He advised Muslims to marry and follow his practice. A study conducted in Bangladesh [34] reported that most females who get married early are Muslims, although Bangladesh is also a Muslim-majority state.

In the current study, most participants mentioned that early marriage has no advantages. However, a few participants referred to some benefits, such as protecting boys and girls, having kids younger, healthier kids, and a younger society. A study conducted in Iran found that adolescent marriage led to better family relationships [30]. Similar beliefs were found in a study in Tamboul town in central Sudan [35].

Most participants of the current study mentioned that early marriage is immature and faces many problems that end in divorce. The kids will be the primary victims of divorce, harmful physiological and physiological effects, financial situations, violence against the wife, cheating on each other, failure to continue their education, and feeling bored after a short period. According to USAID Gender Assessment, young girls are often married to older men, and the age difference reinforces the girl's powerlessness and puts them at higher risk of abuse. Young married girls are more often threatened and beaten and think it might be justified for a husband to beat his wife. Studies from Egypt have revealed that their husbands beat 29% of married young girls. Of these, 41% were beaten during pregnancy [36, 37].

According to a study by UNICEF in 2001, distressed girls and women obliged to get married try to flee from their husbands, while others are abandoned by their husbands. These women are often left with the responsibility of raising children without the husband or family’s financial support [36]. Young married girls face extreme pressure to prove their fertility in their first marriage year. Girls who get married at a young age usually have children early and can have many children. The World Health Organization estimates that the maternal mortality rate is five folds higher for girls aged 14 than for women aged 20. Pregnant adolescents suffer more health issues than older women, especially single girls, who usually obtain less antenatal care. Teen girls are much more liable to anemia than adults, raising pregnancy-related risks and complications. Similarly, they are at higher risk of malnutrition, pregnancy-related hypertension, and eclampsia than women over 20 years [35, 37].

Data from a study in Yemen suggests health risks associated with pregnancy and delivery as the main disadvantages of early marriage, which was why people cited opposition to the practice [38]. Moreover, women who marry at a young age have less decision-making power compared to women who marry in adulthood. Women married as children could not decide on their health care, daily household purchases, household budget, contraception, and visiting family and friends. These women usually have no power over their spouses or in-laws [36].

## Strengths and limitations of the study

This study was done through a qualitative approach which allows the participants to express their thought freely. The considerably large sample size of 16 focus groups is another strength of this study.

This study has a number of limitations. It was conducted only in one governorate (Erbil) in the Kurdistan region, which may not represent the whole Kurdish population. Many factors can limit the validity of a qualitative study. As in the present study, purposive sampling was used; the sample is not random and, therefore, not representative of a bigger population. This means that the validity of the sampling remains open to question. Second, data collection through focus group discussion may lead to problems, such as keeping the subject investigated uniform across focus groups. Each focus group may be different from the others. Third, people are variable; people may say different opinions on different days. Also, one researcher may interpret the dataset of the study in a different way from another. This study is also limited by the lack of generalizability of the finding. The findings of this qualitative study cannot be generalized because we cannot assume that the perspectives and attitudes expressed in one governorate apply to other contexts and groups of people. Such findings can help others consider their situation and learn from the study participants' thoughts, feelings, and actions. All the focus group discussions were conducted in the Kurdish language, so sometimes, finding the exact word in English is difficult to achieve an adequate translation.

## Conclusions

There is a generally negative attitude towards early marriage, and the practice's disadvantages and health and psychological consequences are well recognized in Iraqi Kurdistan Region. However, different social, traditional, economic, educational, and religious factors encourage early marriage in the region. In addition to raising people's awareness about this harmful practice, prevention of early marriage should involve issuing legislation to prohibit it and enforcing such legislation. Further research is recommended in other regional governorates to understand this vital topic.

## Data Availability

The datasets used in this study are available with the corresponding author upon reasonable request.
